# Electrical Threshold Gain Engineering for High-Speed Direct Modulation in Two-Dimensional Semiconductor Laser

**DOI:** 10.1021/acsnano.5c22672

**Published:** 2026-05-13

**Authors:** Zheng-Zhe Chen, Chiao-Yun Chang, Hsiang-Ting Lin, Min-Hsiung Shih

**Affiliations:** † Research Center for Applied Sciences (RCAS), 38017 Academia Sinica, Taipei 11529, Taiwan; ‡ Department of Physics, National Taiwan University, Taipei 10617, Taiwan; § Department of Electrical Engineering, 34880 National Taiwan Ocean University, Keelung 20224, Taiwan; ∥ Department of Electrical and Systems Engineering, 6572 University of Pennsylvania, Philadelphia, Pennsylvania 19104, United States; ⊥ Department of Photonics and Institute of Electro-Optical Engineering, National Yang Ming Chiao Tung University, Hsinchu 30010, Taiwan; # Department of Photonics, National Sun Yat-Sen University, Kaohsiung 80424, Taiwan

**Keywords:** laser modulation, high-speed modulator, suspended transition-metal dichalcogenide, threshold gain tuning, microdisk cavity

## Abstract

Lasers are essential optical modulation sources because of their narrow line width and high coherence. Two-dimensional transition-metal dichalcogenides (TMDCs) exhibit strong exciton binding energy and high material gain and are promising candidates for use in compact, low-threshold semiconductor lasers. Although their intrinsically short exciton lifetimes imply faster modulation compared with bulk semiconductors, no direct TMDC laser modulator has yet been realized. This paper presents a high-speed, room-temperature direct modulator based on a threshold-gain-tunable monolayer tungsten disulfide (WS_2_) microdisk laser. In this modulator, gate voltage can be tuned to modulate the intensity of the lasing output through both carrier density variation and threshold gain control, achieving 50% greater modulation depth compared with normal spontaneous emission. Electrical tuning simultaneously affects the carrier density, dielectric environment, and optical confinement between the WS_2_ monolayer and the cavity. Radiofrequency measurements revealed a 3 dB intensity modulation bandwidth exceeding 120 MHz. Overall, these results demonstrate the feasibility of high-speed direct optical modulation with TMDC lasers, creating opportunities for the development of compact, energy-efficient optoelectronic systems.

## Graphical abstract

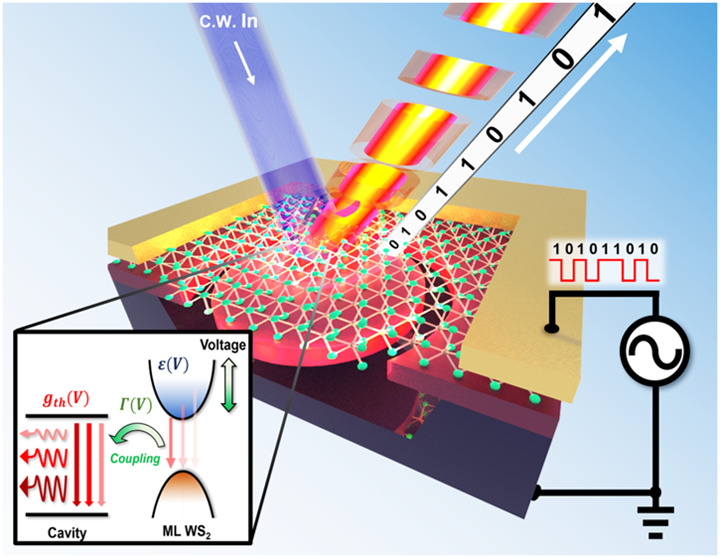

## Introduction

The development of next-generation optoelectronic devices increasingly depends on high-speed operation and device miniaturization. Two-dimensional (2-D) transition-metal dichalcogenides (TMDCs) have attracted increasing attention because of their excellent properties, including atomic-scale thickness, direct bandgaps, and strong exciton binding energies.^1–6^ In particular, monolayer (ML) TMDCs stand out as promising gain media for compact optoelectronic devices, including light-emitting diodes, photodetectors, and low-threshold semiconductor lasers.^7–17^ TMDCs can be flexibly integrated into various optical cavities, enabling the creation of compact lasers with narrow line widths, low thresholds, and excellent coherence.^8–23^

Lasers are key components in optical communication systems because of their spectral purity and long coherence time.^24–27^ Optical modulation is essential for characterizing and controlling laser behavior in practical applications. Although direct modulation has been extensively studied in conventional laser diodes,^28–31^ in which the intensity of lasing is controlled through drive current modulation, it has rarely been applied to TMDC-based lasers. Direct modulation offers advantages over external modulation, including structural simplicity, low energy consumption, and high integration potential.^32–34^ Despite the ultrashort exciton lifetimes of TMDCs,^35–38^ which potentially enable faster modulation capabilities compared with traditional bulk semiconductors, previous studies have primarily focused on external modulators incorporating 2-D materials in waveguides for index or loss tuning.^39–43^ A direct optical modulator leveraging the gain of TMDC laser has yet to be developed.

For high-speed optical communication, key performance requirements include narrow line widths for dense channel multiplexing, long coherence times for stable signal propagation, and fast carrier dynamics enabling high-speed modulation. In this paper, we present a high-speed, room-temperature direct modulator based on an ML tungsten disulfide (WS_2_) microdisk laser that meets these requirements. In this modulator, the WS_2_ laser output can be modulated by tuning the gate voltage gate voltage through threshold gain adjustment, thereby enabling rapid on/off switching. Before modulation measurements, we confirmed lasing behavior through light-in, light-out (L–L) curves, spectral peak narrowing, and coherence analysis. We observed that the modulation of lasing intensity arose from both carrier density variation and changes in the optical confinement factor between the WS_2_ ML and the cavity. Therefore, we evaluated the modulation performance of the proposed device through time-correlated single-photon counting (TCSPC) and radiofrequency (RF) frequency response measurements, and we observed a 3 dB bandwidth exceeding 120 MHz. We further evaluated modulator performance through eye diagram measurement. Our results highlight the potential of TMDC-based lasers as high-speed, compact optical modulators suitable for future optoelectronic integration.

## Results and Discussion

As shown in Figure 1a, to achieve high-speed modulation of a 2-D semiconductor laser at room temperature, a suspended silicon nitride (SiN_*x*_) microdisk cavity was fabricated on a highly doped p^+2^-silicon substrate fully covered with ML WS_2_, which served as the gain material. In this design, the optical properties of WS_2_ were tuned through a back gate voltage (*V*_g_). Detailed device fabrication processes are described in the Methods section. Figure 1b displays a scanning electron microscopy image of the microdisk cavity with ML WS_2_ attached to the surface. The diameter of the microdisk used was approximately 4.3 μm, with a surrounding air gap of 0.5 μm. After ML WS_2_ grown by chemical vapor deposition was carefully transferred onto the microdisk and suspended over the air gap, the 2-D material was suspended to improve radiative efficiency and enhance the optical mode overlap, as demonstrated in our previous study.^21,44^ Next, the performance of the WS_2_ microdisk laser was evaluated using power-dependent photoluminescence (PL) measurements. The fabricated device included a 450 nm continuous-wave (CW) laser was used as the excitation source, focused at the air gap of the microdisk through a 100× lens. Figure 1c presents PL spectra with a pumping power density of 30 W/cm^2^, 2.2 kW/cm^2^, and 3 kW/cm^2^. Although the intrinsic properties of whisper-gallery modes may induce multiple cavity modes within the gain wavelength range, our analysis primarily focuses on the cavity mode peak near 647 nm, corresponding to an azimuthal number *m_z_* = 18 (TE_1,18_ mode; Figure S1).

**Figure 1:**
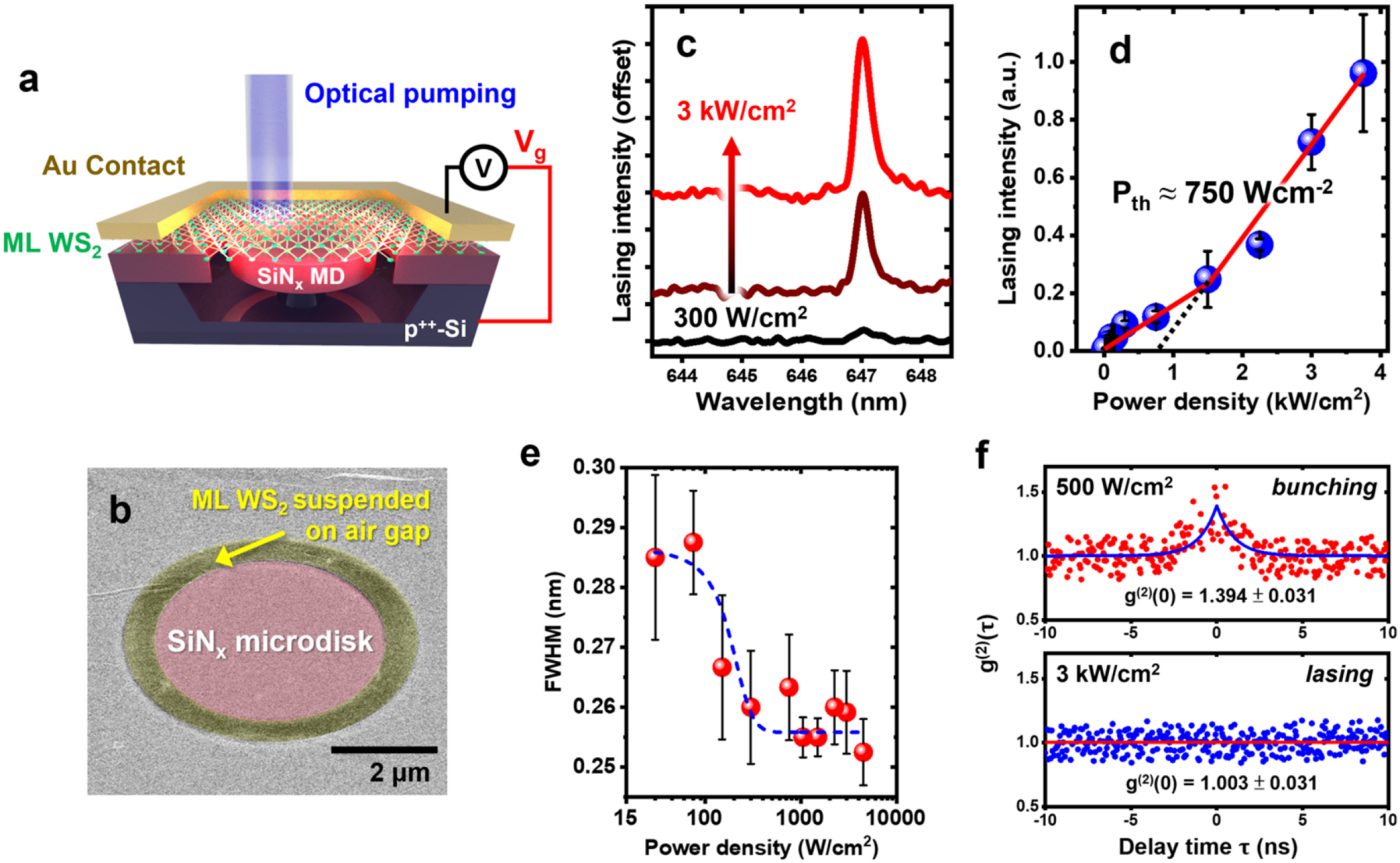
(a) Schematic of a WS_2_ microdisk lasing modulator featuring electrical tuning. ML WS_2_ was attached on top of the SiN_*x*_ microdisk cavity and surrounded by a gold contact. (b) Scanning electron microscopy image of the ML WS_2_ microdisk laser. The microdisk diameter and air gap width were 4.3 μm and 500 nm, respectively. The microdisk and air gap region are depicted in pink and yellow, respectively, and are fully covered with ML WS_2_. (c) Lasing spectra for pumping power density ranging from 30 to 3000 W/cm^2^. (d) L–L curve for laser peak intensity as a function of power density. The extended dashed line indicates the pumping threshold (P_th_) at approximately 750 W/cm^2^. (e) FWHM of lasing peak as a function of power density. A clear line width narrowing indicates the onset of lasing. The blue dashed curve represents the fit of line width narrowing behavior. (f) Second-order coherence function measurements for lasing (cavity) mode at power densities below (red, 500 W/cm^2^) and above (blue, 3 kW/cm^2^) the pumping threshold, revealing photon’s bunching and lasing behavior, respectively. The solid dots represent statistical raw data, and the solid lines represent the Siegert function fitting results.

Figure 1d displays the corresponding L–L curve, demonstrating a typical change in intensity slope from spontaneous to stimulated emission. The threshold power density (*P*_th_) was approximately 750 W/cm^2^, consistent with the line width narrowing behavior illustrated in Figure 1e. The full width at half-maximum (FWHM) of the lasing peak decreased to approximately 0.25 nm as the pumping exceeded the threshold power density, resulting in an experimental quality factor (*Q*) of 2550. In addition, the narrow line width of the microdisk laser, attributed to confined whisper-gallery modes in the cavity, indicating high spectral purity, underscoring the device’s potential for use in the development of multichannel communication systems. To characterize the temporal coherence properties of the ML WS_2_ laser, a typical Hanbury Brown-Twiss interferometer setup was established for second-order photon correlation function *g* ^(2)^(τ) measurements, as depicted in Figure S2 and outlined in the Methods section. The function can be expressed as follows

$$g^{\left(2\right)}\left(\tau \right)=\frac{\left\langle n\left(t\right)n\left(t+\tau \right)\right\rangle }{\left\langle n\left(t\right)\right\rangle \left\langle n\left(t+\tau \right)\right\rangle }$$

where *n*(*t*) is the photon number in the time function. As shown in Figure 1f, *g* ^(2)^(τ) in the time delay function τ led to a statistically significant difference in pumping power below and above the threshold. A Plank’s thermal bunching distribution was observed below the threshold (500 W/cm^2^), whereas a Poissonian photon statistics were observed above the threshold (3 kW/cm^2^). In this figure, the dots represent raw data, and the solid lines represent the fit results with the Siegert relation

$$g^{\left(2\right)}\left(\tau \right)=1+A\exp\left(-\frac{2\left|\tau \right|}{\tau_{c}}\right)$$

where τ_c_ is the temporal coherence of emission. The equal-time second-order photon correlation function at zero time delay *g* ^(2)^(0) was extracted for comparison. When the power density exceeded the threshold (from 500 to 3000 W/cm^2^), the value of *g* ^(2)^(0) decreased significantly from 1.394 to 1.003, indicating our device’s typical coherent lasing performance.

To achieve threshold gain tuning of the ML WS_2_ microdisk laser through electrical control, various gate voltages were applied to bare materials to test several optical properties. Because of their subnanometer thickness and direct bandgap, ML TMDC materials have been widely studied for their unique electrically tunable optical properties.^45–53^ Given the structure of our laser device, a control sample without a microdisk cavity was fabricated to test the voltage-tuning PL of the WS_2_ material (Figure S3a). The back gate voltage (*V*_g_) was continuously adjusted across a wide range, and the PL of the material exhibited pronounced wavelength shifts and intensity changes. The carrier density and Fermi level of the material were directly tuned through bias voltage, ultimately affecting the carriers’ recombination pathways between excitons and trions. When the gate voltage was adjusted from −60 to +100 V, the PL spectrum revealed a distinct wavelength redshift and intensity drop (Figure S3b). In this configuration, 200 nm thick dielectric SiN_*x*_ served as a capacitance layer, the applied bias between the top electrode and back gate modulates the carrier concentration inside the ML semiconductor WS_2_ and consequently adjusted the PL intensity magnitude.

Figure 2a depicts the overall voltage-induced changes to the microdisk WS_2_ laser threshold gain. Voltage-induced modulation of material carrier density simultaneously tuned the refractive index and dielectric constant (ε), as demonstrated in previous studies.^54–56^ To estimate the change in the dielectric function of our system, we conducted conventional reflection measurements in the presence of different gate voltages (*V*_g_, Figure S4a). We primarily focused on wavelengths near the WS_2_ bandgap to extract the complex dielectric function (ε_1_, ε_2_) through Kramers–Kronig constrained analysis (Figure S4b).^54–56^ We fitted the complex dielectric function ε = ε_1_ + *i*ε_2_ through a contribution sum of multiple Lorentz oscillators. We noticed that the real part of the dielectric function (ε_1_) exhibited a noticeable reduction from 25.9 to 24.7 as the gate voltage was increased to +30 V (Figure S4c).

**Figure 2:**
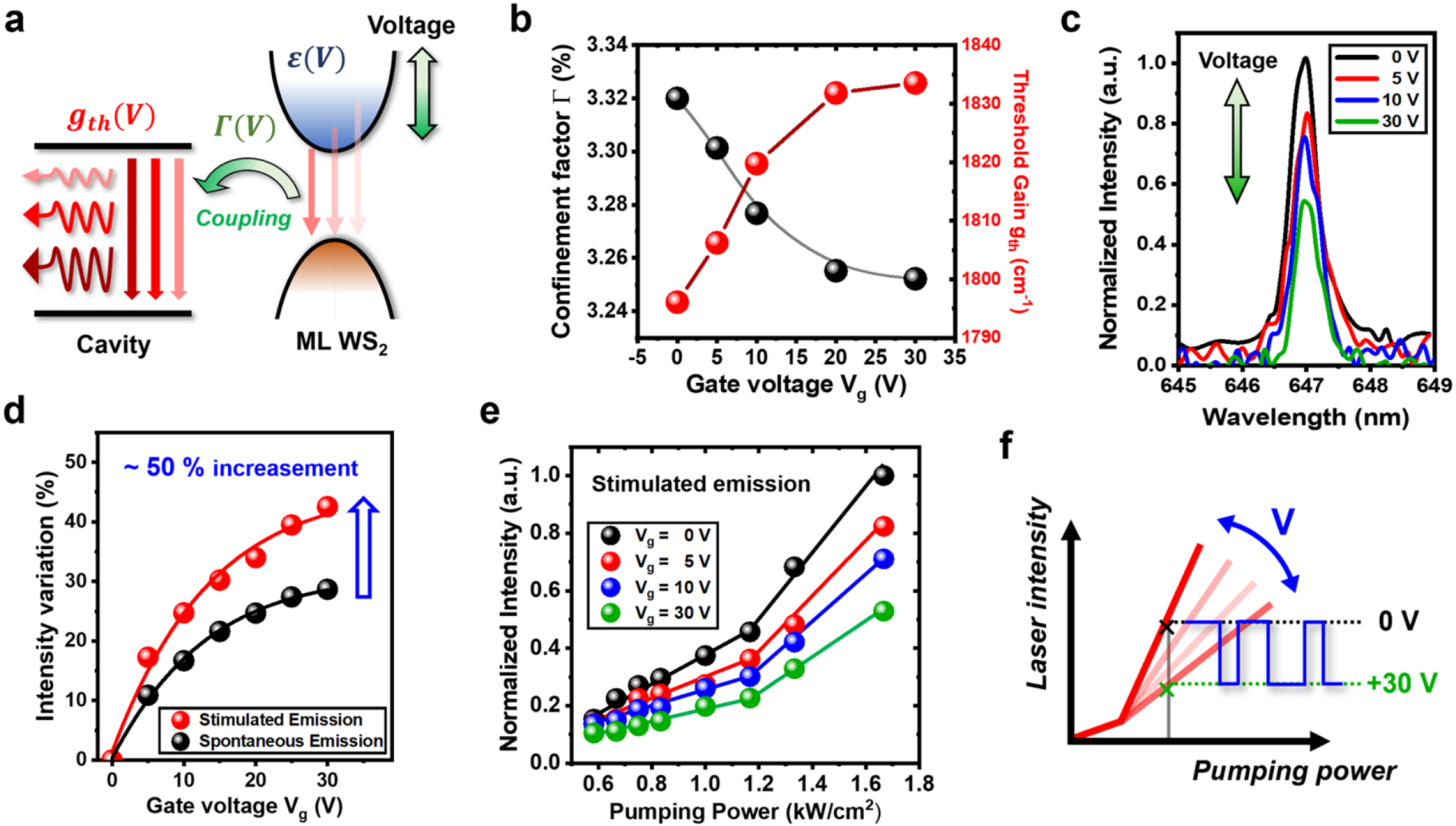
(a) Schematic illustrating gate-voltage control of the lasing intensity. The applied voltage (*V*) modulates the dielectric function (ε) of ML WS_2_, which alters the confinement factor (Γ) and the coupling between the gain medium and the cavity, thereby tuning the threshold gain (*g*_th_) and the resulting lasing intensity. (b) Change in the confinement factor (black) and threshold gain (red) as a function of gate voltage. (c) Lasing spectra with gate voltage (*V*_g_) tuning from 0 to 30 V and with pumping power density fixed at 3 kW/cm^2^. And a near 50% of modulation depth is observed under a 30 V bias. (d) Intensity variation for lasing (red) and spontaneous (blue) emission as a function of gate voltage. Under the same gate voltage modulation, the lasing signal shows a modulation depth about 50% larger than that of spontaneous emission. (e) L–L curve of lasing peak intensity at differing gate voltages. The solid lines represent linear fit, demonstrating on-switching behavior. Under different *V*_g_, different turn-on slope reveals the intrinsic change of the threshold gain. (f) Schematic illustrating how voltage-induced gain tuning can be used as a switch for optical modulation. With fixed optical pumping, rapid modulation of *V*_g_ leads to fast changes in the lasing intensity with a large modulation depth.

The optical confinement factor (Γ) quantifies the overlap between the gain medium and the cavity mode, which can be expressed as follows

$$\Gamma =\frac{\int_{\Omega }\varepsilon_{{\mathrm{WS}}_{2}}\left(r\right){\left|E\left(r\right)\right|}^{2}dr}{\int_{-\infty }^{+\infty }\varepsilon (r){\left|E\left(r\right)\right|}^{2}dr}$$

where ε_WS_2__(*r*) is the dielectric constant of ML WS_2_, *E*(*r*) is the electric field, and Ω is the region of the active medium. In this study, the fitting results presented in Figure S4c were combined with finite element method analysis to calculate the optical confinement factor (Γ) at different magnitudes of gate voltage (*V*_g_, Figure 2b). Next, the cavity loss was evaluated to estimate the threshold gain (*g*_th_) of the material in the proposed laser device. Thus, we determined the total cavity loss δ_r_ as follows

$$\delta_{r}=\frac{2\pi n_{\mathrm{eff}}}{Q\lambda }$$

where *n*_eff_ is the effective index and *Q* and λ are the quality factor and wavelength of the lasing peak, respectively. When cavity loss meets the lasing threshold, modal gain compensates for this loss

$$\delta_{r}={\Gamma g}_{\mathrm{th}}$$

Thus, the threshold gain (*g*_th_) can be expressed as follows

$$g_{\mathrm{th}}=\frac{2\pi n_{\mathrm{eff}}}{\Gamma Q\lambda }$$

Figure 2b depicts the threshold gain of our laser device in the presence of different gate voltages. The device exhibited considerable tunability, varying by approximately 40 cm^–2^ (from 1796 to 1834 cm^–2^) within a 30 V back gate voltage range. Experimentally, we observed a distinct change in lasing peak intensity with varying back gate voltages (Figure 2c). As the gate voltage *V*_g_ increased, the peak intensity dramatically decreased, reaching almost half its magnitude when *V*_g_ was applied at 30 V because of the gain tuning effect. In the presence of a high bias voltage, the narrow supporting pillar beneath the microdisk may have caused the current to concentrate at the pole site and penetrate through the dielectric layer; therefore, we restricted the maximum tuning range to 30 V to prevent any current leakage or breakthrough. To confirm the contributions of the confinement factor and threshold gain changes rather than intrinsic carrier concentration changes, normalized intensity variation was compared in stimulated (lasing) and spontaneous emission (Figure 2d). At all gate voltages applied, the relative intensity variation in the lasing regime was approximately 1.5 times larger than that in spontaneous emission, likely because of the effects of carrier concentration and threshold gain change. Consequently, the larger intensity variation in the lasing mode indicated higher tunability (modulation depth), exceeding that of conventional light-emitting diodes for practical optical modulation.

To understand the electrical-switching behavior of stimulated emission, L–L curves were examined as a function of different gate voltages (Figure 2e). We primarily focused on pumping power near the laser threshold to acquire clear data. As indicated by the L–L curve, the gate voltage tuned both the carrier concentration and the threshold gain, which directly affected the on-switching slope and threshold power. At *V*_g_ = 0 V (black solid line), the laser device exhibited more favorable laser properties, as indicated by the steeper slope, whereas higher gate voltages produced a more gradual slope and increased the threshold, which is compatible with the threshold gain tendency presented in Figure 2b. For comparison, we also examined the L–L curve of spontaneous PL emission (Figure S5). The major differences in performance illustrated in Figures 2e and S5 confirm the contribution of threshold gain tuning rather than carrier concentration change to the laser signal. Despite the many advantages of TMDC lasers, including their compactness, low threshold, and relatively short photon lifetime,^35–38^ their practical implementation remains limited. The unique optical properties of TMDC lasers suggest that these devices could be promising candidates for high-speed optical modulators. Therefore, given the threshold gain tuning capacity of our microdisk WS_2_ laser device, gate voltage can be used to electrically switch the laser on and off. As shown in Figure 2f, in the presence of fixed pumping power, voltage and lasing peak intensity serve as the input and output, respectively. Gate voltage can be switched between 0 and +30 V, effectively transforming our laser device into a binary (on/off) optical modulator.

To better understand the temporal modulation performance of our laser device, a continuous on/off modulation measurement was conducted. Figure 3b illustrates the continuous voltage-switching behavior of lasing intensity with gate voltages switched between 0 and 30 V. We focused on the lasing peak wavelength to minimize the effects of spontaneous emission, and we observed no clear intensity degradation over multiple on/off cycles. The high stability of our WS_2_ laser modulator at normal pressure and room temperature underscores its promise for practical applications. Figure 3c compares intensity behavior at different voltage-switching levels. At different gate voltage magnitudes, all lasing intensities exhibited stable and reversible behavior. In contrast to *V*_g_ = 30 V (green solid line), lower gate voltages (10 and 5 V) demonstrated reduced modulation depths, consistent with the results displayed in Figure 2d.

**Figure 3:**
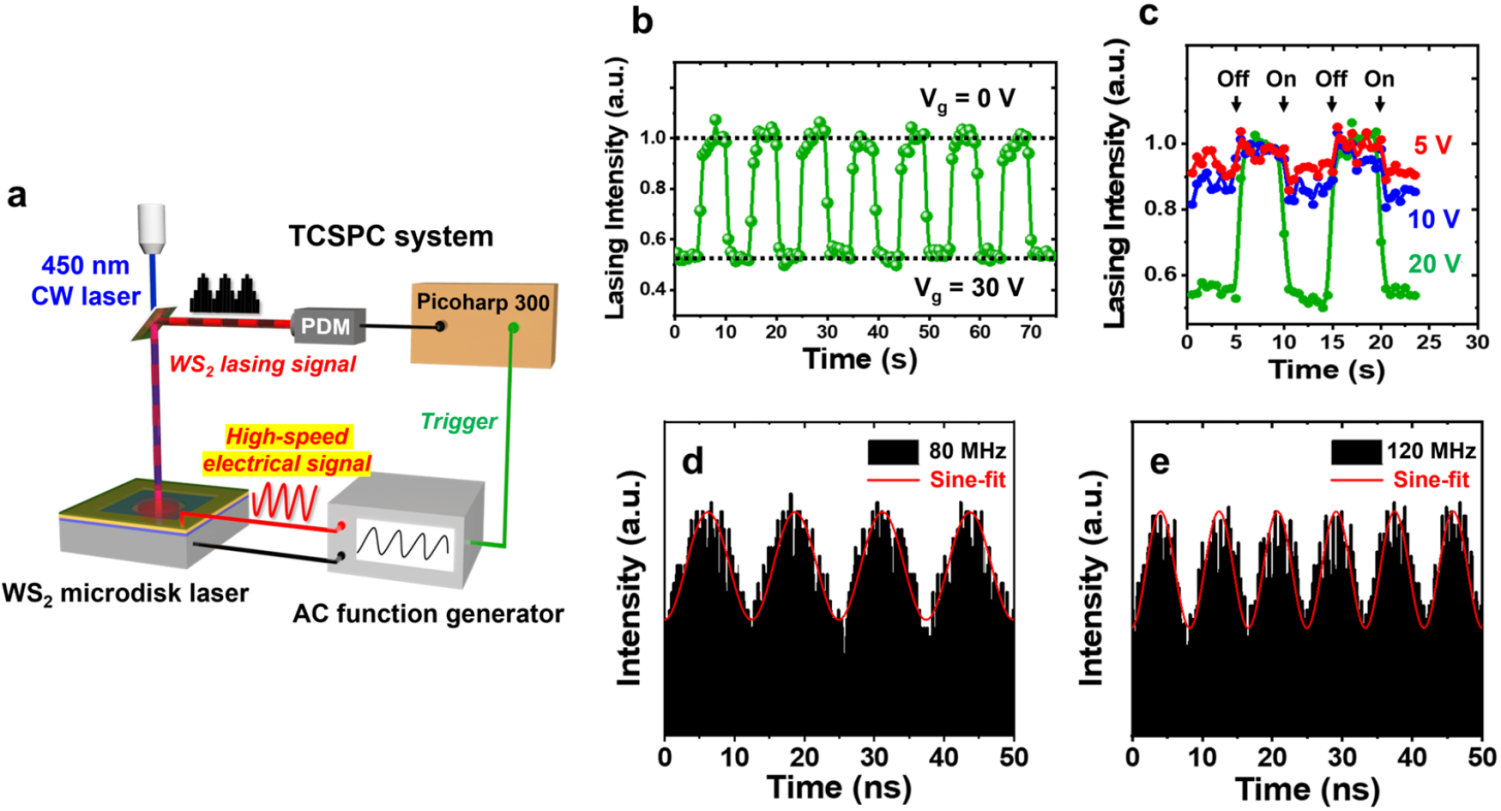
(a) Schematic of the TCSPC system setup for high-speed modulation measurements. PDM: photon-detection module. (b) Continuous tuning of lasing peak intensity at gate voltages of 0 and 30 V, showing an average 45% of modulation depth. (c) Modulation of lasing intensity at *V*_g_ = 5 V (red), 10 V (blue), and 30 V (green). (d, e) High-speed TCSPC measurement histograms of lasing signals at frequency modulation values of 80 and 120 MHz. The solid black bars represent statistical modulation signal results, and the red solid lines represent sine function fitting.

To characterize the modulation speed of our laser modulator, we employed a TCSPC system to characterize high-speed modulation.^57^ Figure 3a depicts a schematic of the measurement setup. The aforementioned pumping source was fixed at a power density of 3 kW/cm^2^ to generate a lasing signal. Next, the device was supplied with an alternating current (AC) generated by a function generator (up to 120 MHz) between the top electrode and back gate electrode rather than a fixed bias voltage. A high-speed sinusoidal AC signal was then applied at an amplitude of 8 V (limited to 120 MHz by the function generator) to achieve high-speed threshold gain tuning. Before the modulated lasing signal was collected by a photon detection module (PDM), the total emission from the laser device was filtered through diffraction grating inside a charge-coupled device to block out the spontaneous emission wavelength (not depicted here for simplicity). A Picoharp 300 event timer was then used to quantify the photon signal, synchronized with the AC signal from the function generator. Because of the narrow line width of the lasing peak, the modulated lasing signal was weak and had a limited photon count, which could be directly resolved by the TCSPC system without additional amplification. Figure 3d[Fig fig3],e display the TCSPC histograms of an electrically modulated lasing signal at 80 and 120 MHz of high-speed voltage switching, respectively. Although 120 MHz was the maximum sine wave frequency achievable with our function generator, the clear intensity contrast in Figure 3e indicates that 120 MHz does not appear to represent the intrinsic limit of our microdisk WS_2_ laser modulator. Based on the current device configuration, the modulation bandwidth is primarily limited by the RC time delay (∼2.1 ns). A simple estimation suggests that the intrinsic modulation speed could reach a few hundred MHz, exceeding the experimentally observed value. And we believe further improvements in material quality and device design are expected to reduce the RC delay and enable higher modulation speeds.

To further validate our device’s high-speed modulation response, the modulated lasing signal was collected by a photodetector and monitored in real time with an oscilloscope. A clear waveform was observed when the function generator provided a 100 MHz sine wave electrical signal (Figure S6), further confirming the high-speed modulation capability of the laser.

To demonstrate the feasibility of our device in practical optical communication, we conducted standard eye diagram measurements. As shown in Figure 4a, the ML WS_2_ microdisk laser direct modulator was optically pumped using a CW laser and simultaneously driven by a function generator to supply a pseudorandom binary sequence electrical signal (PRBS frequency up to 100 Mbps at 10 *V*_pp_ and a rise/fall time of 3.3 ns) (Figure 4a). The generated lasing signal was then passed through two edge filters before being collected by a visible nanosecond photodetector (Responsivity at 620 nm: 0.4 A/W; rise/fall time <2 ns), which efficiently converted optical intensity into a high-speed electrical signal. The signal was amplified by a radiofrequency amplifier (RF AMP) for eye diagram visualization on an oscilloscope. As depicted in Figure 4b, a well-defined and open eye diagram was obtained at a data rate of 50 Mbps. The corresponding bit error rate (BER) was estimated as 1.75 × 10^–9^, which is considerably below the forward error correction threshold. These results confirm that our WS_2_ laser modulator can support reliable high-speed optical communication. An additional measurement at 100 Mbps is presented in Figure S7. Although four eyes remained distinguishable on the diagram, the signal exhibited increased noise and reduced eye opening, indicating the degradation of performance at higher data rates as a result of signal fluctuation and jitter, which may be mainly attributed to timing-related limitations, including RC delay in the device and the finite rise/fall time of the function generator (∼3.3 ns). Further optimization of both device parameters and high-speed measurement components is expected to improve the modulation performance.

**Figure 4:**
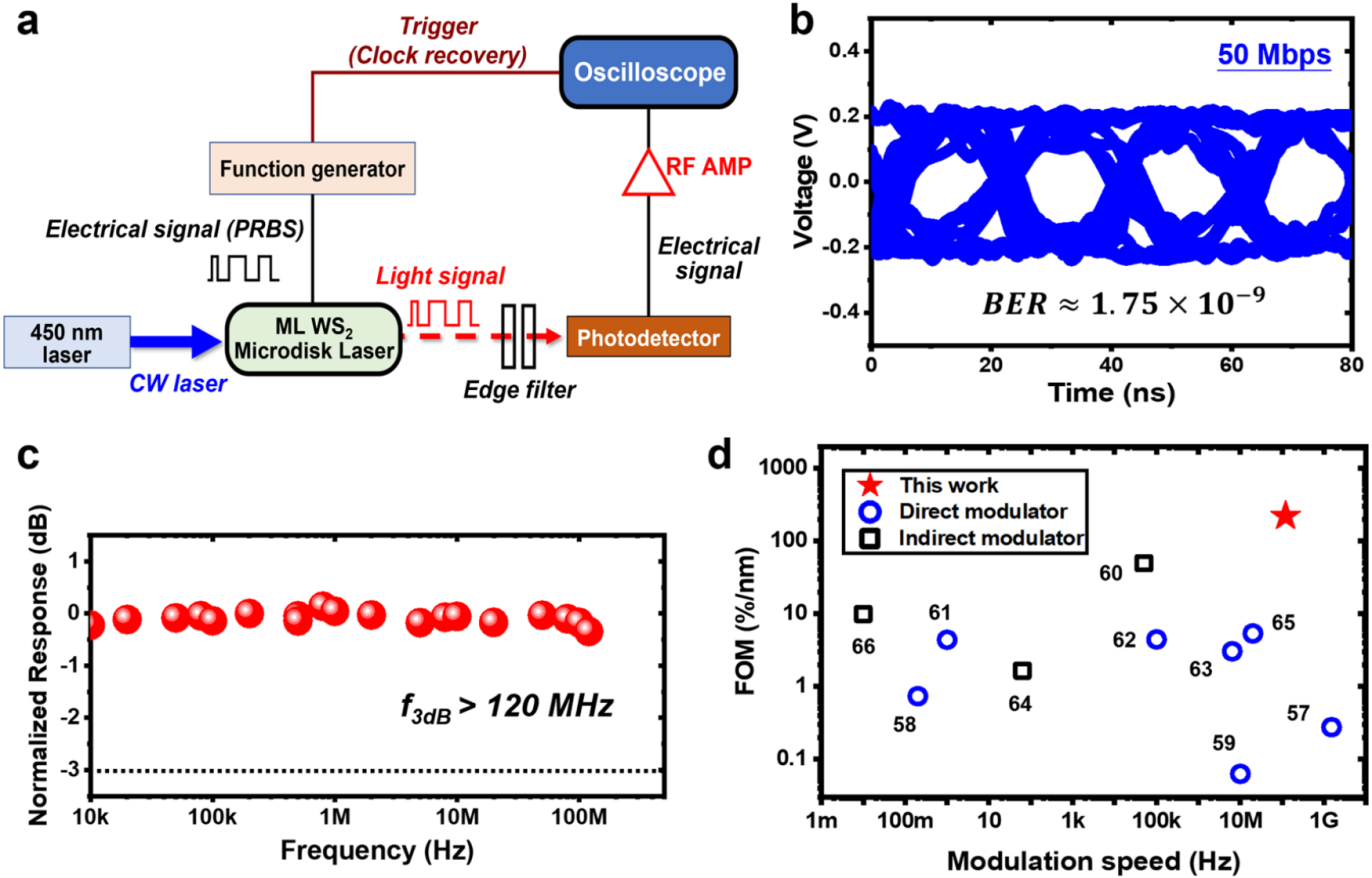
(a) Schematic of the eye diagram measurement setup. PRBS: pseudorandom binary sequence; RF AMP: radiofrequency amplifier. (b) Eye diagram response from the WS_2_ microdisk laser at a data rate of 50 Mbps with bit error ratio (BER) around 1.75 × 10^–9^. (c) Normalized lasing intensity response as a function of modulated frequency. The 3 dB cutoff frequency (*f*_3dB_) was above the 120 MHz limit of our function generator. (d) Benchmark of FOM versus modulation speed for a 2-D material–based EO modulator.

In addition to eye diagram analysis, we measured broadband frequency responses to further characterize the modulation speed of our device and determine the 3 dB cutoff frequency. Briefly, the WS_2_ microdisk laser was optically pumped by a 450 nm CW laser, while an AC function generator simultaneously applied a high-frequency sine wave signal (10 to 120 MHz) to modulate the threshold gain of the laser. The lasing peak was spectrally selected using a spectrometer (CCD) and connected to an oscilloscope through an amplifier to monitor and compare the modulated output with the original input signal. The frequency response results are displayed in Figure 4c. The lasing intensity remained stable up to approximately 50 MHz, with only a minor reduction (approximately 1 dB) observed at 100 MHz. No significant roll-off was observed within the instrument’s measurement range, indicating that the 3 dB cutoff frequency of our device exceeded the instrument limit of 120 MHz (i.e., *f*_3dB_ > 120 MHz). It is worth to note that the III–V semiconductor lasers can reach modulation bandwidths in the tens to hundreds of GHz range (refer the Table S8 in the Supporting Information)^58–63^ which is faster than the speed of 2-D TMDC modulators at current stage. However, lavage on the intrinsically fast exciton dynamics in TMDCs,^64,65^ the modulation speed from 2-D TMDC devices can be much improved by optimizing material growth quality and device design in the future.

Three key parameters are essential for practical optical communication: a narrow line width, which enables denser channel spacing; a large modulation depth, which improves the signal-to-noise ratio; and a high modulation speed, which enables higher data throughput. To better understand how our device compares with other 2-D material–based electrooptic (EO) modulators, including TMDCs and graphene, we reviewed related studies that have been recently published.^57,64–74^ To compare spectral purity and modulation capability, we proposed a figure of merit (FOM) defined as follows

$$\mathrm{FOM}\;\left(\%/\mathrm{nm}\right)=\frac{m\mathrm{odulation}\;\mathrm{depth}\;\left(\%\right)}{\mathrm{FWHM}\;\left(\mathrm{nm}\right)}$$

This FOM represents the product of modulation depth and spectral purity, providing a compact performance indicator.

Figure 4d provides a summary of the FOM values of recent EO modulators, including our proposed device, as a function of modulation speed. Our analysis included direct and indirect EO modulators for which modulation depth, speed (or rise/fall time), and spectral characteristics have been reported. Benchmark comparison clearly demonstrated the competitive modulation speed and superior FOM of our WS_2_ laser modulator compared with previously reported 2-D material–based EO modulators, highlighting its promise for application in high-performance optical communication systems. A table in Session S9 of the Supporting Information show the characteristics of the TMDC-based direct electro-optic modulators and representative high-speed photodetectors reported in the literatures.^57,67,69,70,75,76^ The comparison indicates that the modulation device in the study exhibits an ultrafast modulation speed and an intrinsically narrow emission line width. These features provide clear advantages for high-speed data transmission and high channel density in future optical communication systems.

## Conclusion

This study demonstrates the potential of TMDC lasers for application as high-speed optical direct modulators at room temperature. In this study, we constructed an ML WS_2_ microdisk laser capable of rapidly switching its lasing signal on and off through gate voltage modulation. To validate the performance of the proposed device, we first confirmed its laser operation through the on-switching behavior observed in the L–L curve, peak line width narrowing, and second-order coherence *g* ^(2)^ measurements. We adjusted the gate voltage to manipulate the lasing intensity, which was affected by both material carrier density and the confinement factor between WS_2_ and the cavity. We observed a difference of approximately 50% in lasing intensity when the gate voltage was increased from 0 V to +30 V. Moreover, at various gate voltages, stimulated emission consistently exhibited approximately 1.5 times greater modulation depth compared with that observed with spontaneous PL, indicating the combined effects of carrier concentration and threshold gain change. Through continuous temporal measurements and analysis with a TCSPC system, we further confirmed the stability and 120 MHz modulation capability of our laser modulator at room temperature. Finally, through a broadband RF response test, we determined that the 3 dB cutoff frequency of our device exceeded 120 MHz. We also observed that our WS_2_ laser modulator provided comparable modulation speeds to other 2-D material–based EO modulators in addition to a remarkably narrow line width, underscoring its suitability for practical optical communication. Overall, our findings demonstrate the promise of TMDC lasers in achieving high-speed direct optical modulation at room temperature, showing the feasibility for the future development of compact optical communication systems and EO applications.

## Methods

### Device Fabrication

First, a 200 nm-thick Si_3_N_4_ layer was deposited on a high-doped p^+2^-silicon substrate through low pressure chemical vapor deposition (LPCVD). Next, to fabricate the microdisk cavity, we utilized electron beam lithography (EBL) to create the pattern. The electron beam resist poly­(methyl methacrylate) (PMMA) was spin-coated on Si_3_N_4_ for EBL development. After development, the substrate then went under an inductively coupled plasma reactive-ion etching (ICP-RIE) process. The residual resist PMMA was removed with oxygen plasma in the same ICP-RIE. Next the as-etched substrate was immersed in 40 wt % KOH solution at 60 °C to form a suspended microdisk structure through an anisotropic wet etching of the Si substrate.

The ML WS_2_ was then transferred on top of the microdisk and carefully suspended on top of the air gap by PMMA-assisted wet transfer method. A CVD-grown large-area ML WS_2_ flake on a sapphire substrate was first spin-coated with PMMA and heated to 180 °C. The ML WS_2_-sapphire substrate was then immersed in KOH solution to separate the ML WS_2_ from the sapphire substrate and adhered to the upper PMMA layer. Next, the PMMA-ML WS_2_ layer was carefully scooped up and transferred onto the as-fabricated cavity to achieve full coverage. The sample was then baked at 100 °C to remove the residual water. Later, the sample was immersed in acetone to remove the PMMA layer, followed by a thorough rinse with isopropanol alcohol (IPA).

Finally, aligned photolithography development was applied for top contact. A 3-μm-thick photoresist layer was patterned using a laser direct writer for aligned exposure around the device, with an alignment accuracy better than 500 nm. Subsequently, a 50 nm gold electrode was deposited by thermal evaporation followed by a lift-off process.

### Optical Measurement

All the PL spectrum were measured by the home-build confocal-microscope μ-PL system. For PL measurements, a 450 nm wavelength CW diode laser is utilized as source (except the time-resolved PL measurement) and focused by a 100×, N.A.= 0.55 objective lens with spot size around 1 μm. And the PL radiation is collected by the same objective lens and further recorded/analyzed by a spectrograph with thermoelectrically cooled charge-coupled device (CCD). The resolution of spectrum was approximately 0.06 nm. For temporal measurements, like second-order coherence, the signal was collected using a single photon avalanche diode connected with a time-corrected single photon counting (TCSPC) module.

Schematics of the optical setup of second-order coherence function *g* ^(2)^(τ) measurement, which is known as a Hanbury Brown-Twiss (HBT) interferometer system is illustrated in Supporting Information Figure S2. Signal generated from our WS_2_-microdisk cavity is collected by a 100× objective lens and split into two paths by a 50/50 beam splitter. The signals from different paths were then collected respectively by two photon-detection modules (PDM 1&2) and analyzed by a Picoharp 300 time-correlated single photon counting (TCSPC) system. The raw data was further fit with the Siegert relation

$$g^{\left(2\right)}\left(\tau \right)=1+A\exp\left(-\frac{2\left|\tau \right|}{\tau_{c}}\right)$$

where τ_c_ is the temporal coherence of emission.

## Supplementary Material



## Notes

Mode profile; *g* ^(2)^(τ) measurement setup; voltage-tuning PL; voltage-tuning dielectric function analysis; light-in-light-out curve of PL; 100 MHz-modulated intensity profile; 100 Mbps eye diagram; and semiconductor laser modulator benchmarks and TMDC direct EO modulator benchmarks (PDF)
