# Supplementary material for: Electrical Threshold Gain Engineering for High-Speed Direct Modulation in Two-Dimensional Semiconductor Laser
Source: ACS Nano. 2026 May 13;20(20):14543–51. doi: 10.1021/acsnano.5c22672 (PMC13218047; doi:10.1021/acsnano.5c22672)
Supplement: Supplementary file 1 [file nn5c22672_si_001.pdf]

Supporting Information for

Electrical Threshold Gain Engineering for High-Speed Direct Modulation in Two-Dimensional Semiconductor Laser

Zheng-Zhe Chen[1,2], Chiao-Yun Chang[3], Hsiang-Ting Lin[4], and Min-Hsiung Shih[1,5,6]*

[1]Research Center for Applied Sciences (RCAS), Academia Sinica, Taipei 11529, Taiwan.

[2]Department of Physics, National Taiwan University, Taipei 10617, Taiwan.

[3]Department of Electrical Engineering, National Taiwan Ocean University, Keelung 20224, Taiwan.

[4]Department of Electrical and Systems Engineering, University of Pennsylvania, PA 19104, USA

[5]Department of Photonics and Institute of Electro-Optical Engineering, National Yang Ming Chiao Tung University, Hsinchu 30010, Taiwan.

[6]Department of Photonics, National Sun Yat-sen University, Kaohsiung 80424, Taiwan.

*Corresponding author. Email: mhshih@gate.sinica.edu.tw

S1. Mode profile of microdisk cavity with monolayered WS_2

Fig. S1 depicts the top-view mode profile from the WGM of the magnetic field (H_z) of our monolayered WS_2 on the gap microdisk, which was obtained using the FEM calculation. The black circles represent the inner and outer edges of the air gap, where the cavity WGM was mainly distributed. A clear $TE_{1,18}$ mode exhibited and confined at the air gap site, forming a high Q performance at around 463.4 THz (646.9 nm).

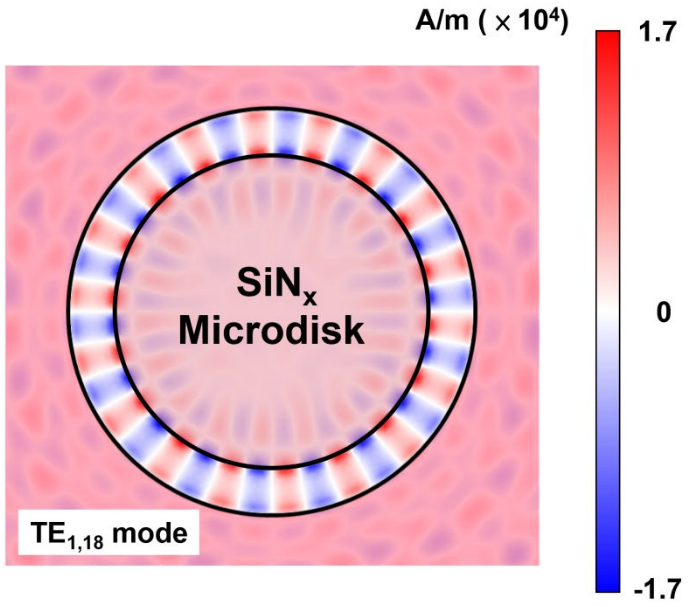

Fig. S1. Mode profiles of horizontal magnetic field (H_z) distribution from monolayer WS_2 on a gap microdisk. Black solid lines represent SiN_x edge. Pink part depicts the SiN_x area.

S2. Second-order coherence function $g^{(2)}(\tau)$ measurement setup

Fig. S2 illustrates the schematics of the optical setup of $g^{(2)}(\tau)$ measurement, which is known as a Hanbury Brown-Twiss (HBT) interferometer system. The EL/lasing signal generated from our WS_2-microdisk cavity is collected by a 100 × objective lens. The lasing light was purified through two edge filters in wavelength and split into two paths by a 50/50 beam splitter. The signals from different paths were then collected respectively by two photon-detection modules (PDM1&2) and analyzed by a Picoharp 300 time-correlated single photon counting (TCSPC) system.

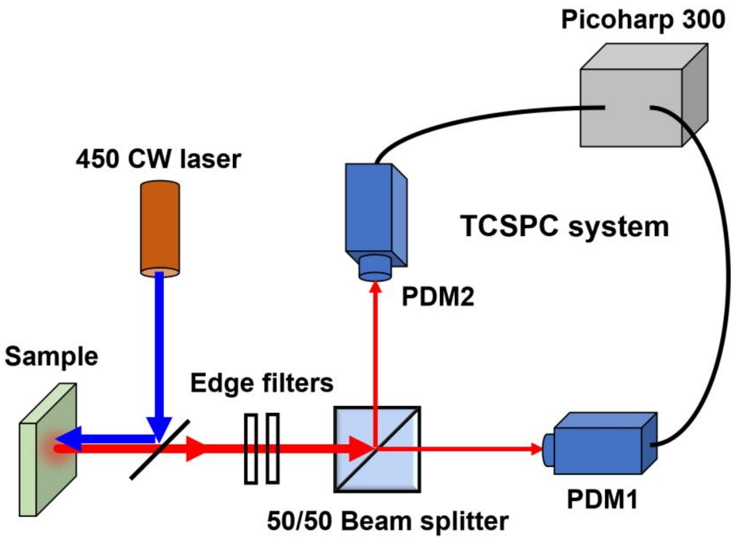

Fig. S2. Schematic of the setup of second coherence function $g^{(2)}(\tau)$ measurements.

S3. Voltage-tuning photoluminescence of ML WS_2

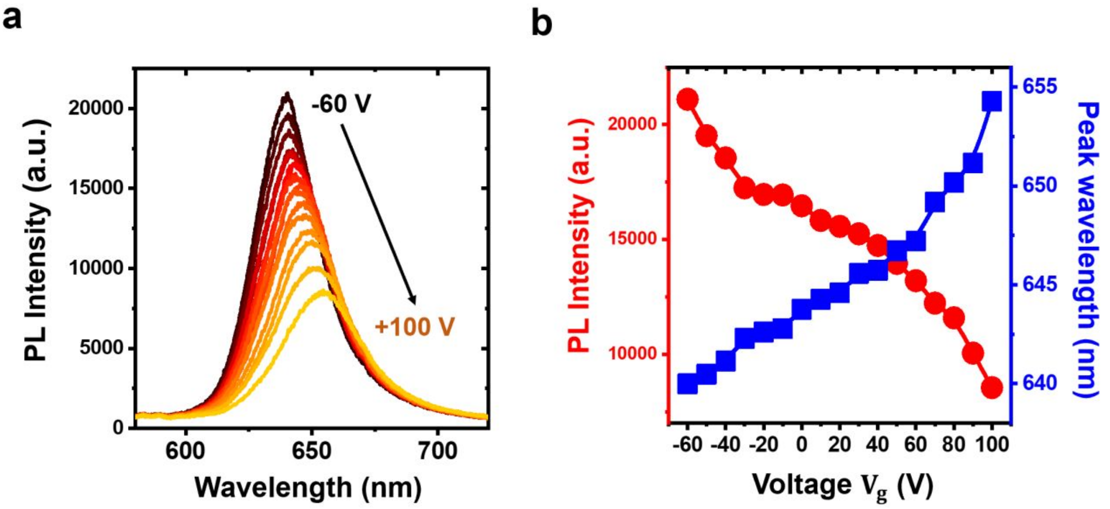

Fig. S3. a PL spectra of bare ML WS_2 with gate voltage tuning from -60 to +100 V. **b** ML WS_2 PL intensity (red) and peak wavelength (blue) in function of gate voltage V_g.

S4. Voltage-tuning dielectric function analysis of ML WS_2

We mainly focus on wavelengths near WS_2's bandgap, we estimated the material's dielectric constants $(\varepsilon_1, \varepsilon_2)$ through Kramers–Kronig constrained analysis based on the voltage-dependent reflection spectra we measured (Fig. S4a). The complex dielectric function $\varepsilon = \varepsilon_1 + i\varepsilon_2$, was fitted by a contribution sum of multiple Lorentz oscillators,

$$\boldsymbol{\varepsilon_1 + i\varepsilon_2 = \varepsilon_\infty + \sum_j \frac{f_j}{E_j^2 - E^2 - iE\gamma_j}}$$

with ε_∞: background dielectric constant; f_j: oscillator strength; E_j: resonant energy and γ_j: linewidth of oscillator.

Fitted results are revealed in Fig. S4b. Real part of dielectric function (ε_1) exhibited a noticeable reduction from 25.9 to 24.7 as the gate voltage was increased to +30 V (Fig. S4c), which is reliable for further voltage-dependent confinement factor and threshold gain calculation (Fig. 2b).

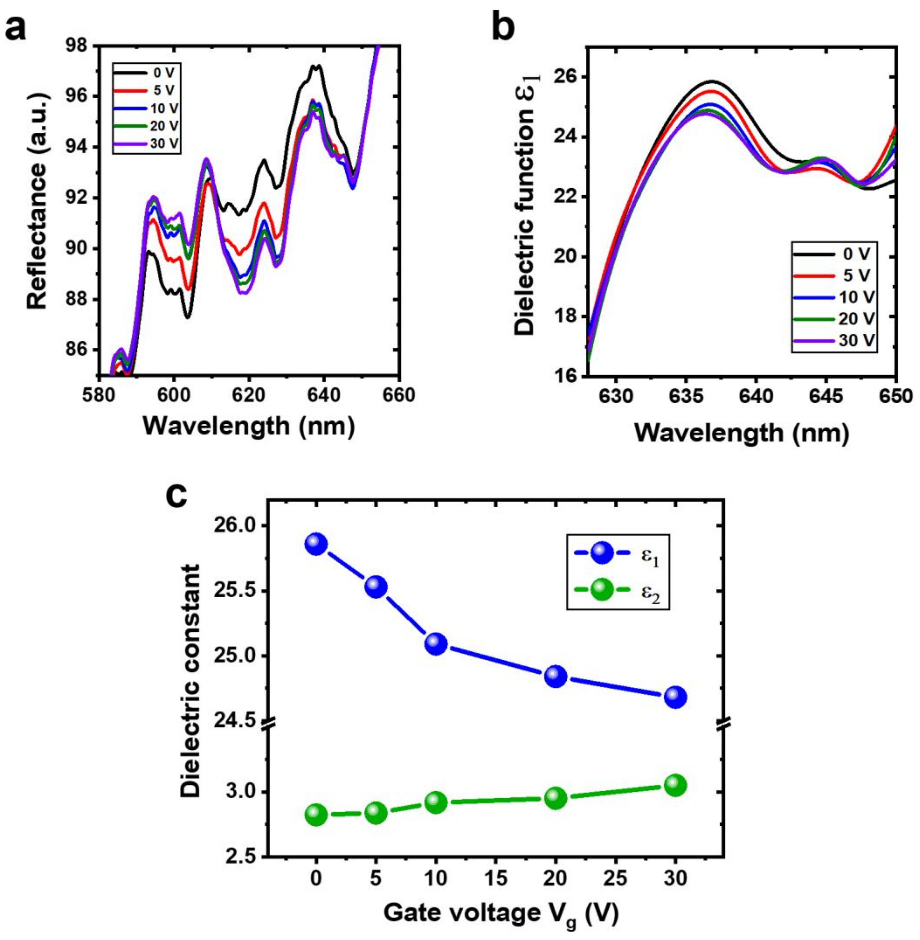

Fig. S4. a Reflection spectra of ML WS_2 near bandgap with gate voltage tuning from 0 to 30 V. **b** Fitted results of real part dielectric function near bandgap under different gate voltage. spectra of bare ML WS_2 with gate voltage tuning from -60 to +100 V. **c** Value of real (blue) and imaginary (green) part of dielectric function in function of applied gate voltage.

S5. Light in-light out curve of spontaneous PL emission

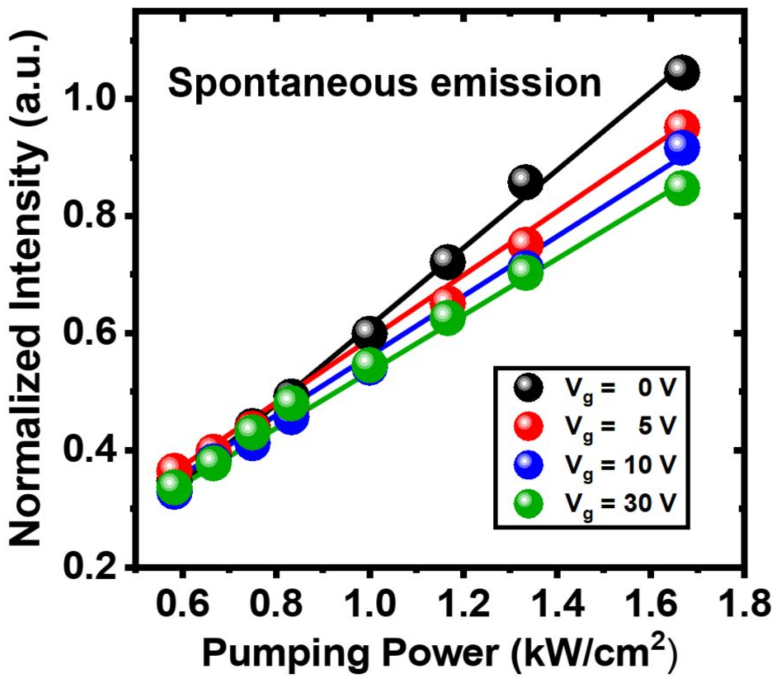

Fig. S5. Light in-light out curve of spontaneous emission intensity with different level of gate voltage applying. Solid lines are the linear fitting to clarify the tendency.

S6. 100 MHz-modulated lasing intensity profile from oscilloscope

Besides TCSPC system, our modulated lasing signal can also be detected from a photodetector and monitored in an oscilloscope. A high-speed 100 MHz modulating signal shows a clear profile from oscilloscope (Fig. S6).

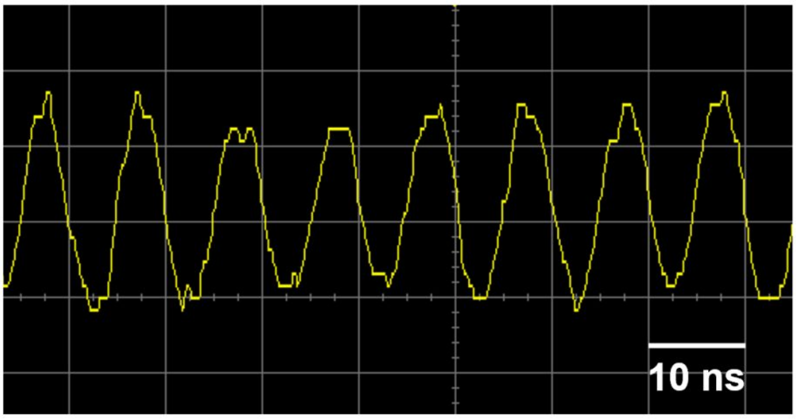

Fig. S6. Real-time lasing intensity signal profile from oscilloscope at 100 MHz modulation speed. Time scale bar is 10 ns.

S7. Eye diagram under 100 Mbps data rates

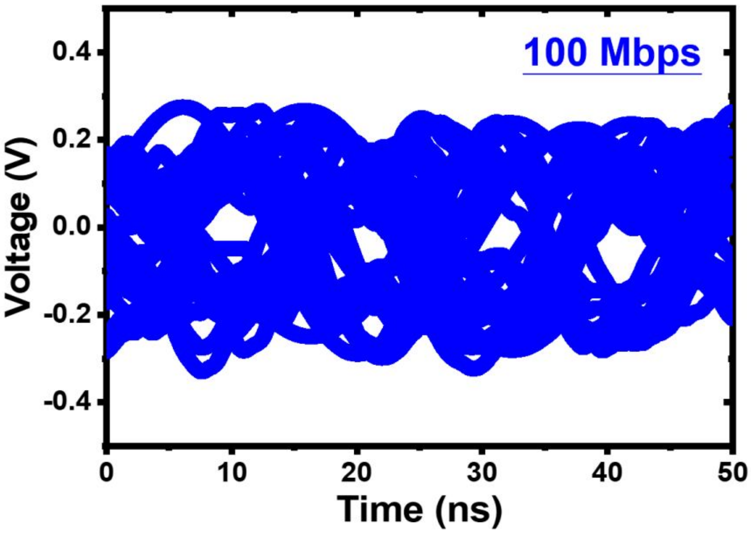

Fig. S7. 100 Mbps data rates eye diagram response from WS_2 microdisk laser.

S8. High-speed semiconductor laser modulator benchmarks

In the session, Table S8 shows the modulation speeds from the 2-D TMDC microcavity laser in the study and several III-V semiconductor compact lasers under the directly modulation operation. The micro-lasers with the III-V gain materials had demonstrated modulation bandwidths exceeding tens to hundreds of GHz (S1-6). Although the modulation speed of the 2-D TMDC laser in this work (~120 MHz) is currently lower than the speed of the mature III-V semiconductor lasers, the performances of 2-D TMDC laser could be much improved in the future by optimizing the material epitaxial growth and engineering device operations.

Importantly, the intrinsic exciton lifetime in monolayer TMDCs has been reported to be in the picosecond to sub-picosecond regime (S7), suggesting that the ultimate modulation speed is not fundamentally limited by the material itself. With further improvements in material quality, device design, and electrical optimization, the modulation bandwidth is expected to be significantly increased toward the GHz regime or beyond.

Laser type	Modulation speed / 3 dB bandwidth	References
**ML TMDC-microdisk laser**	**> 120 MHz**	**This work**
InGaN-based laser diode	> 30 Gbps	(S1)
III-V DFB laser	60 GHz	(S2)
MQW DFB laser	108 GHz	(S3)
Two-κ DBR laser	65 GHz	(S4)
VCSELs	> 200 GHz	(S5)
InAsP/InP nanowire-photonic crystal laser	10 Gbps	(S6)

Table. S8. Table of the high-speed directly modulation characteristics from the laser devices with the different gain materials and cavity structures.

S9. High-speed TMDC-based direct electro-optic modulator benchmarks

In the session, shown in the Fig. S9, a comparison table has been organized to benchmark the 2-D TMDC laser in the study and the previously reported 2-D TMDC-based direct modulators (S8-11). From the perspective of future optical communication applications, key performance metrics, including the emission full-width at half-maximum (FWHM), modulation speed, and modulation depth, are systematically listed in the table. In addition, the modulation speed is further benchmarked against several representative high-speed photodetectors reported in the literature (S12, 13).

The results indicate the reported device in the study exhibits an ultrafast modulation speed and an intrinsically narrow emission linewidth, and will benefit to future high-speed data transmission and high channel density in optical communication integrated systems.

Material type	Emission FWHM (nm)	Speed (MHz)	Light modulation depth (%)	Device type	References
**WS_2**	**0.25**	**120**	**45**	**Direct EO modulator (Laser)**	**This work**
WSe_2	~10	0.1	~81.5	Direct EO modulator (with cavity)	(S8)
WS_2	20	10	~60	Direct EO modulator	(S9)
WSe_2	22	-	95.8	Direct EO modulator	(S10)
WS_2	36.3	800	~50	Direct EO modulator (LED)	(S11)
WSe_2	-	22	-	Photodetector	(S12)
WSe_2	-	1.9	-	Photodetector	(S13)

Table. S9. Table of the modulation characteristics (emission FWHM, modulation speed, and modulation depth) from the reported 2-D TMDC-based modulator sand photodetectors.

References:

(S1) Hu, J.; Gu, Z.; Jia, H.; Yang, Z.; Li, Z.; Wu, J.; Sun, L.; Sun, A.; Huang, O.; Xia, C.; et al. InGaN-based high-speed mini laser diode surpasses PAM-4 visible light links by over 30 Gbps. *Science China Information Sciences* **2026**, *69* (3). DOI: 10.1007/s11432-025-4581-8.

(S2) Diamantopoulos, N.-P.; Fujii, T.; Yamaoka, S.; Nishi, H.; Takeda, K.; Tsuchizawa, T.; Segawa, T.; Kakitsuka, T.; Matsuo, S. 60 GHz Bandwidth Directly Modulated Membrane III-V Lasers on SiO2/Si. *Journal of Lightwave Technology* **2022**, *40* (10), 3299-3306. DOI: 10.1109/jlt.2022.3153648.

(S3) Yamaoka, S.; Diamantopoulos, N.-P.; Nishi, H.; Nakao, R.; Fujii, T.; Takeda, K.; Hiraki, T.; Tsurugaya, T.; Kanazawa, S.; Tanobe, H.; et al. Directly modulated membrane lasers with 108 GHz bandwidth on a high-thermal-conductivity silicon carbide substrate. *Nature Photonics* **2020**, *15* (1), 28-35. DOI: 10.1038/s41566-020-00700-y.

(S4) Matsui, Y.; Schatz, R.; Che, D.; Khan, F.; Kwakernaak, M.; Sudo, T. Low-chirp isolator-free 65-GHz-bandwidth directly modulated lasers. *Nature Photonics* **2020**, *15* (1), 59-63. DOI: 10.1038/s41566-020-00742-2.

(S5) Lindemann, M.; Xu, G.; Pusch, T.; Michalzik, R.; Hofmann, M. R.; Zutic, I.; Gerhardt, N. C. Ultrafast spin-lasers. *Nature* **2019**, *568* (7751), 212-215. DOI: 10.1038/s41586-019-1073-y.

(S6) Takiguchi, M.; Yokoo, A.; Nozaki, K.; Birowosuto, M. D.; Tateno, K.; Zhang, G.; Kuramochi, E.; Shinya, A.; Notomi, M. Continuous-wave operation and 10-Gb/s direct modulation of InAsP/InP sub-wavelength nanowire laser on silicon photonic crystal. *APL Photonics* **2017**, *2* (4). DOI: 10.1063/1.4977927.

(S7) Palummo, M.; Bernardi, M.; Grossman, J. C. Exciton radiative lifetimes in two-dimensional transition metal dichalcogenides. *Nano Lett* **2015**, *15* (5), 2794-2800. DOI: 10.1021/nl503799t.

(S8) Liu, C. H.; Clark, G.; Fryett, T.; Wu, S.; Zheng, J.; Hatami, F.; Xu, X.; Majumdar, A. Nanocavity Integrated van der Waals Heterostructure Light-Emitting Tunneling Diode. *Nano Lett* **2017**, *17* (1), 200-205. DOI: 10.1021/acs.nanolett.6b03801.

(S9) Zhu, G.; Zhang, L.; Li, W.; Shi, X.; Zou, Z.; Guo, Q.; Li, X.; Xu, W.; Jie, J.; Wang, T.; et al. Room-temperature high-speed electrical modulation of excitonic distribution in a monolayer semiconductor. *Nat Commun* **2023**, *14* (1), 6701. DOI: 10.1038/s41467-023-42568-w.

(S10) Ye, T.; Zhou, B.; Liu, Z.; Li, Y.; Shen, H.; Ning, C. Z.; Li, D. Room-Temperature Exciton-Based Optoelectronic Switch. *Small* **2021**, *17* (5),

e2005918. DOI: 10.1002/smll.202005918.

(S11) Kwak, D.; Paur, M.; Watanabe, K.; Taniguchi, T.; Mueller, T. High-Speed Electroluminescence Modulation in Monolayer WS2. *Advanced Materials Technologies* **2021**, *7* (5). DOI: 10.1002/admt.202100915.

(S12) Zhang, Y.; Shen, W.; Wu, S.; Tang, W.; Shu, Y.; Ma, K.; Zhang, B.; Zhou, P.; Wang, S. High-Speed Transition-Metal Dichalcogenides Based Schottky Photodiodes for Visible and Infrared Light Communication. *ACS Nano* **2022**, *16* (11), 19187-19198. DOI: 10.1021/acsnano.2c08394.

(S13) Zhang, Y.; Ma, K.; Zhao, C.; Hong, W.; Nie, C.; Qiu, Z. J.; Wang, S. An Ultrafast WSe(2) Photodiode Based on a Lateral p-i-n Homojunction. *ACS Nano* **2021**, *15* (3), 4405-4415. DOI: 10.1021/acsnano.0c08075.
